# The complete mitochondrial genome of the Korean endemic earthworm *Amynthas deogyusanensis* (Clitellata: Megascolecidae)

**DOI:** 10.1080/23802359.2022.2161839

**Published:** 2023-01-08

**Authors:** Jachoon Koo, Yong Hong

**Affiliations:** a Division of Science Education and Institute of Fusion Science, College of Education, Jeonbuk National University, Jeonju, Republic of Korea; b Department of Agricultural Biology, College of Agriculture & Life Sciences, Jeonbuk National University, Jeonju, Republic of Korea

**Keywords:** *Amynthas deogyusanensis*, mitochondrial genome, Megascolecidae, phylogeny

## Abstract

The Korean endemic earthworm *Amynthas deogyusanensis* Hong and James, 2001 (Clitellata: Megascolecidae) is found in the forest area of Deogyu Mountain, South Korea. In this study, the complete mitochondrial genome (mitogenome) of *A. deogyusanensis* was sequenced, assembled, and annotated. The mitogenome of *A. deogyusanensis* is a circular DNA molecule, consisting of 15,257 bp with an A + T content of 67.9%. It contains 13 protein-coding genes, two ribosomal RNA genes, 22 transfer RNA genes, and one non-coding region (control region). Phylogenetic analysis suggested that the family Megascolecidae is a monophyletic group with full support, whereas the genus *Amynthas* is non-monophyletic with the genera *Metaphire* and *Duplodicodrilus*.

*Amynthas deogyusanensis* Hong and James, 2001 is an earthworm endemic species to South Korea. The genus *Amynthas* Kinberg, 1867 is known to comprise more species than any other genus of the *Pheretima* complex (Sims and Easton 1972). *Amynthas* is the largest genus in family Megascolecidae and is one of the most abundant and diverse genus found in Korea and East Asia. Deogyu Mountain National Park located in Jeollabuk-do (central Korea), preserves the sub-alpine ecosystem. *Amynthas deogyusanensis*, belonging to the family Megascolecidae, is a species first discovered in the Deogyu Mountain, South Korea (Hong and James 2001). The body length, width, and segments of the species range from 102 to 110 mm, 5 to 5.7 mm, and 104 to 106 mm, respectively (Hong and James 2001). The male pore region of this species is unique and can be easily distinguished from the other endemic South Korean species (Figure 1).

**Figure 1.**
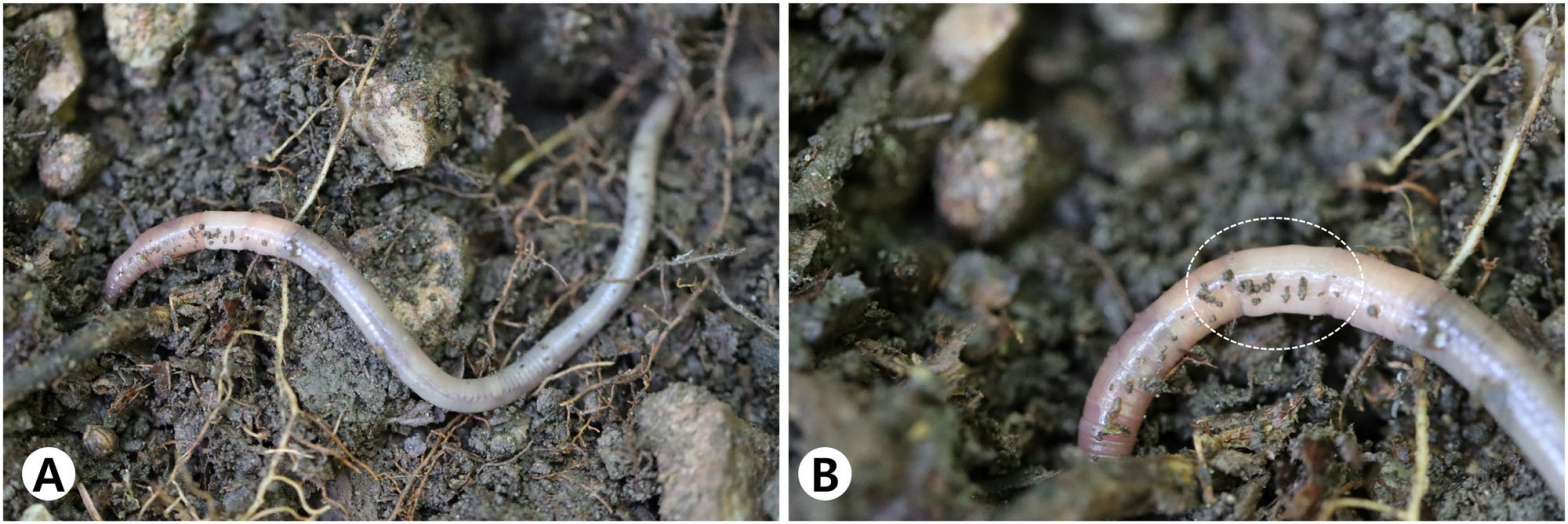
Clitellate of *Amynthas deogyusanensis*. (A) The dorsal view; (B) the clitellum annular XIV–XVI. The photograph from Deogyu Mountain, South Korea by Yong Hong on 2 August 2020.

The specimens of *A. deogyusanensis* were collected from Deogyu Mountain, Jeollabuk-do, South Korea (35°52′12.78″ N, 127°48′49.36″ E; 1296 m) on 2 August 2020. A specimen was deposited at Jeonbuk National University (Yong Hong, yonghong@jbnu.ac.kr) under the voucher number JBNU0004.

Total DNA was isolated from a single specimen using QIAamp DNA Mini Kit (Qiagen, Hilden, Germany) and a sequencing library was constructed using Illumina TruSeq DNA Nano Library Prep Kit (Illumina Inc., San Diego, CA). The mitogenome sequence was generated by paired-end (2 × 150 bp) sequencing using Illumina HiSeq-X platform (San Diego, CA). The raw reads were assembled using SPAdes version 3.13.0 (Bankevich et al. 2012) based on the GenBank-registered reference mitogenome sequence of the earthworm *A. pectiniferus* (GenBank accession number: KT429018). Mitogenome annotation was conducted using MitoZ version 2.3 (Meng et al. 2019) and manually curated based on BLAST searches in the National Center for Biotechnology Information (NCBI) database. To explore the evolutionary relationships and phylogenetic position of *A. deogyusanensis*, the available mitogenome sequences of the 27 species in the Megascolecidae family were collected from the NCBI database (Boore and Brown 1995; Wang et al. 2015; Zhang et al. 2016a, 2016b, 2019; Hong et al. 2017). Mitogenome sequences from the Lumbricidae and Moniligastridae families were used as outgroups. Nucleotide sequences of 13 protein-coding genes (PCGs), 22 transfer RNA genes (tRNAs), and two ribosomal RNA genes (rRNAs) from each mitogenome were aligned using the clustal omega tool in Geneious Primer 2021, and the aligned sequences were concatenated into a dataset. The phylogenetic tree was constructed by the Bayesian inference (BI) method using MrBayes v3.2.7 (Ronquist et al. 2012). Markov chain Monte Carlo analysis was performed for 1,000,000 generations (the average standard deviation of split frequencies was 0.007). The first 25% of the tree corresponding to the ‘burn-in’ period was discarded and the remaining parts of the tree were used to construct the majority-rule consensus tree.

The complete mitogenome of *A. deogyusanensis* is a circular DNA molecule consisting of 15,257 bp with 13 PCGs, 22 tRNAs, two rRNAs, and one major non-coding control region (Figure 2). The arrangement of mitochondrial genes of *A. deogyusanensis* is identical to that of other available mitogenomes of megascolecids species (Boore and Brown 1995; Wang et al. 2015; Zhang et al. 2016a, 2016b, 2019). We observed that all 13 PCGs start with an ATG codon, which is typical for invertebrate mitochondrial PCGs. Seven PCGs end with an complete stop codons, TAA and TAG, and six PCGs end with an incomplete stop codon, T. The A + T content of the whole mitogenome is 67.9%, which is similar to that found in the megascolecid species (61.6–67.2%). A 679-bp fragment of the putative control region is located at the junction between *trnR* and *trnH*.

**Figure 2.**
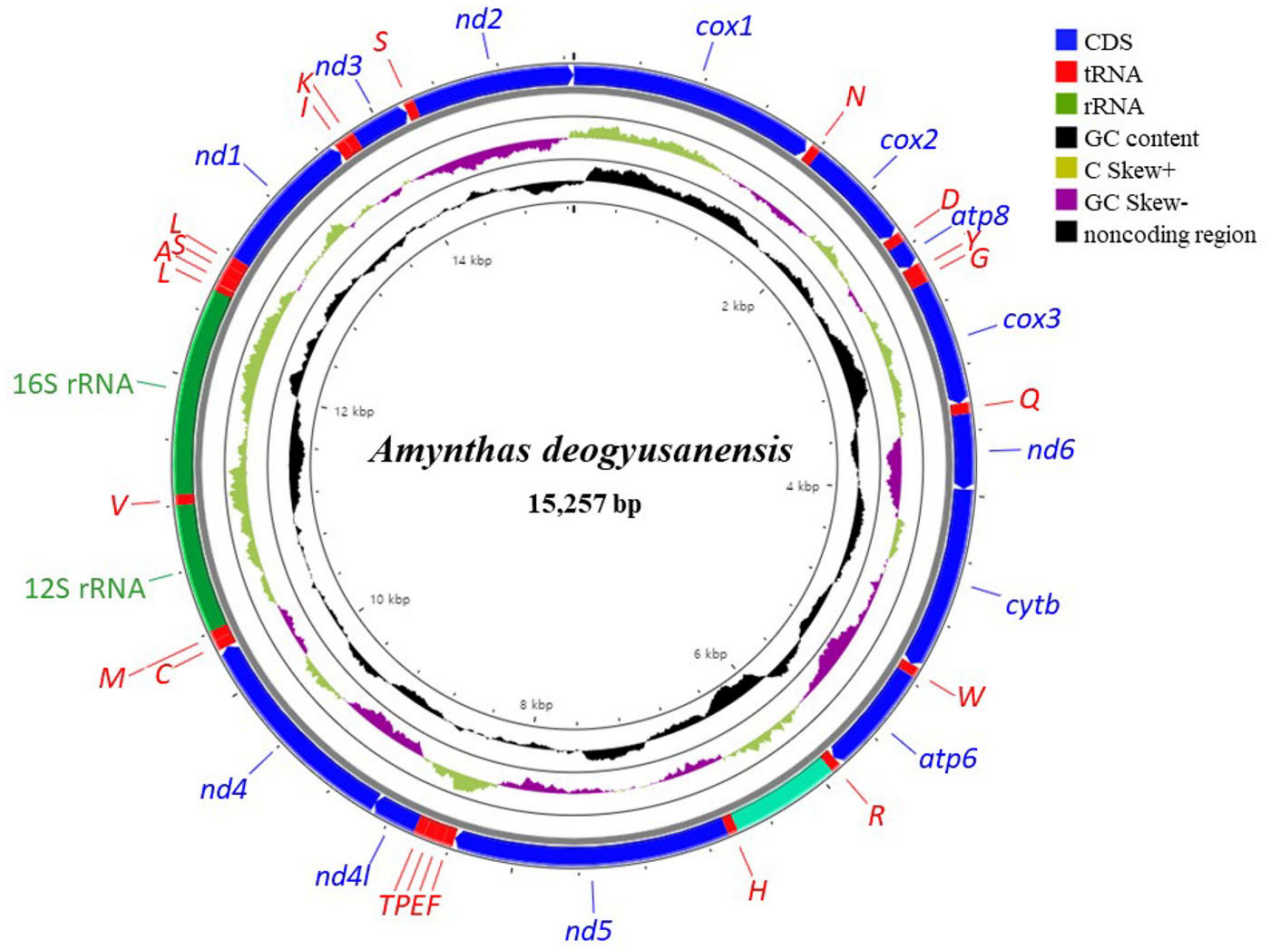
Circular sketch map of the *Amynthas deogyusanensis* mitogenome. The mitogenome map was generated with CGView server (http://cgview.ca). Each transfer RNA gene is represented by a one-letter amino acid code. Different colors represent different gene blocks.

Phylogenetic analysis suggested that *A. deogyusanensis* is a sister group of *A. yunoshimensis*, *M. hilgendorfi*, *A. jiriensis*, *A. spatiosus*, *A. hupeiensis*, *A. instabilis*, and *A. seungpanensis* in the family Megascolecidae (Figure 3). This relationship presents high nodal support in BI analysis. Additionally, the family Megascolecidae presents a monophyletic group with the highest nodal support, whereas *Amynthas* appears as a non-monophyletic group with the genera *Metaphire* and *Duplodicodrilus* (Hong et al. 2017; Zhang et al. 2019).

**Figure 3.**
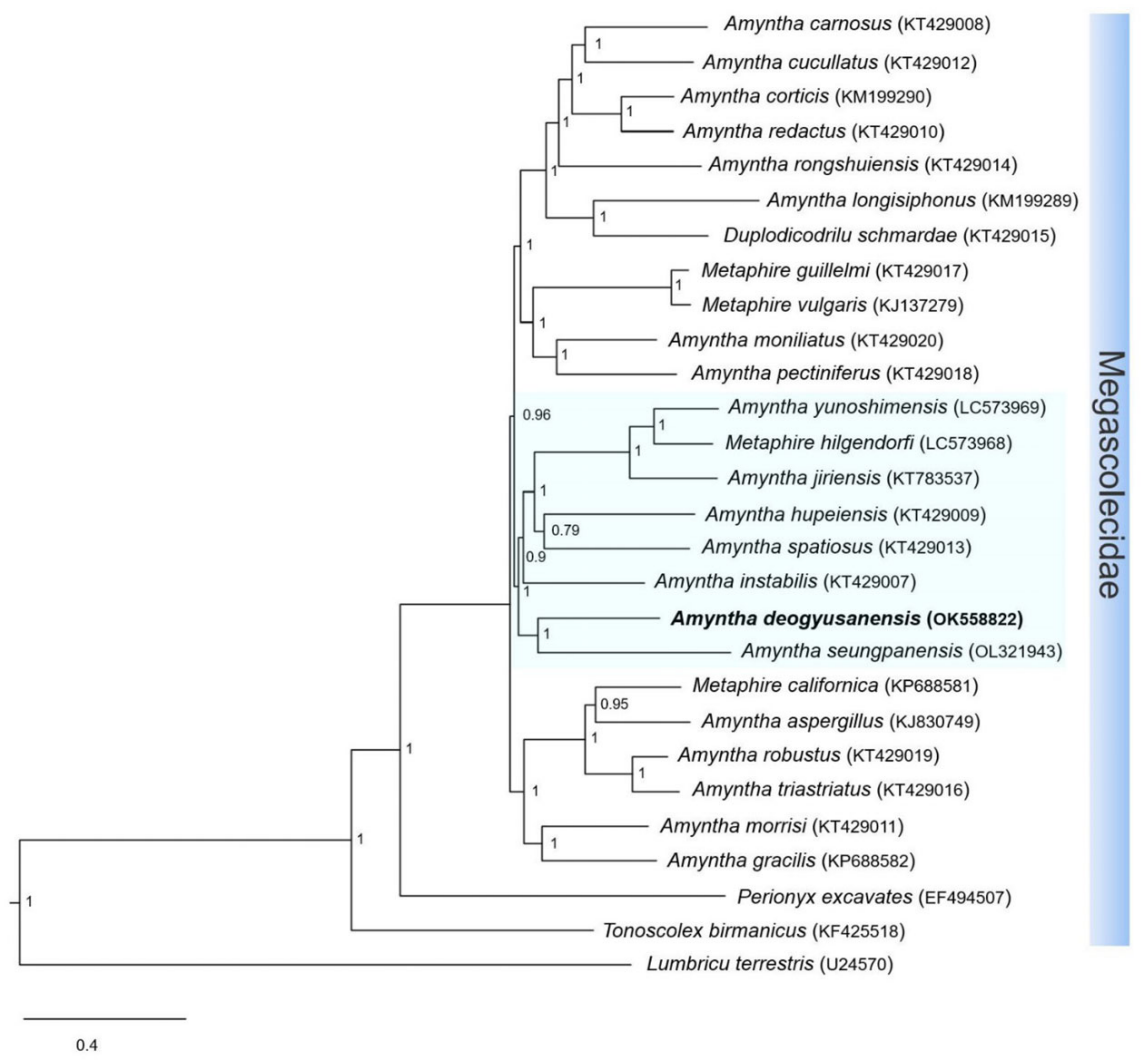
Phylogenetic tree of the 27 species of the Megascolecidae family. Phylogenetic analysis was done using Bayesian inference (BI) method, based on whole mitogenome sequences, excluding the non-coding region. The numbers at each node specify Bayesian posterior probabilities (BPP) by BI. The scale bar indicates the number of substitutions per site. *Lumbricus terrestris* (Boore and Brown 1995) is used as the outgroup. GenBank accession numbers are mentioned next to species names.

## Data Availability

The genome sequence data that support the findings of this study are openly available in GenBank of NCBI at https://www.ncbi.nlm.nih.gov/nuccore/OK558822, under the accession no. OK558822. The associated BioProject, SRA, and Bio-Sample numbers are PRJNA769829, SRR16507334, and SAMN22374388, respectively.
